# A Peripheral CB1R Antagonist Increases Lipolysis, Oxygen Consumption Rate, and Markers of Beiging in 3T3-L1 Adipocytes Similar to RIM, Suggesting that Central Effects Can Be Avoided

**DOI:** 10.3390/ijms21186639

**Published:** 2020-09-10

**Authors:** Rebecca L. Paszkiewicz, Richard N. Bergman, Roberta S. Santos, Aaron P. Frank, Orison O. Woolcott, Malini S. Iyer, Darko Stefanovski, Deborah J. Clegg, Morvarid Kabir

**Affiliations:** 1 Sports Spectacular Diabetes and Obesity Wellness and Research Center, Cedars-Sinai Medical Center, Los Angeles, CA 90048, USA; RPaszkiewicz@mednet.ucla.edu (R.L.P.); Richard.Bergman@cshs.org (R.N.B.); santosrds1@gmail.com (R.S.S.); aaronpfrank@gmail.com (A.P.F.); Orison.Woolcott@gmail.com (O.O.W.); malini.s.iyer@gmail.com (M.S.I.); 2 School of Veterinary Medicine, University of Pennsylvania, Philadelphia, PA 19104, USA; sdarko@vet.upenn.edu; 3 The College of Nursing and Health Professions, Drexel University, Philadelphia, PA 19104, USA; djc387@drexel.edu

**Keywords:** beige adipocytes, lipolysis, CB1R antagonist, mitochondrial function, obesity

## Abstract

With the increased prevalence of obesity and related co-morbidities, such as type 2 diabetes (T2D), worldwide, improvements in pharmacological treatments are necessary. The brain- and peripheral-cannabinoid receptor 1 (CB1R) antagonist rimonabant (RIM) has been shown to induce weight loss and improve glucose homeostasis. We have previously demonstrated that RIM promotes adipose tissue beiging and decreased adipocyte cell size, even during maintenance on a high-fat diet. Given the adverse side-effects of brain-penetrance with RIM, in this study we aimed to determine the site of action for a non-brain-penetrating CB1R antagonist AM6545. By using in vitro assays, we demonstrated the direct effects of this non-brain-penetrating CB1R antagonist on cultured adipocytes. Specifically, we showed, for the first time, that AM6545 significantly increases markers of adipose tissue beiging, mitochondrial biogenesis, and lipolysis in 3T3-L1 adipocytes. In addition, the oxygen consumption rate (OCR), consisting of baseline respiratory rate, proton leak, maximal respiratory capacity, and ATP synthase activity, was greater for cells exposed to AM6545, demonstrating greater mitochondrial uncoupling. Using a lipolysis inhibitor during real-time OCR measurements, we determined that the impact of CB1R antagonism on adipocytes is driven by increased lipolysis. Thus, our data suggest the direct role of CB1R antagonism on adipocytes does not require brain penetrance, supporting the importance of focus on peripheral CB1R antagonism pharmacology for reducing the incidence of obesity and T2D.

## 1. Introduction

The prevalence of obesity is growing worldwide and is associated with several metabolic diseases including type 2 diabetes (T2D) and cardiovascular disease [1,2,3]. White adipose tissue (WAT) is the main organ in control of energy homeostasis in mammals, which facilitates storage of excess energy as triglycerides. Brown adipose tissue (BAT), on the other hand, is metabolically active and dissipates energy as heat which is used to maintain body temperature. In addition to classic BAT, the development of brown-like, or beige, adipocytes from white adipocytes leads to improved metabolic homeostasis within WAT via dissipation of extra energy as heat [4]. The beiging of WAT can be induced under various treatments such as cold exposure or β-adrenergic stimulation [5,6]. Obesity is now classified as a disease due to the fact that it has been associated and correlated with hypertrophy, stress, and inflammation, conditions which, at their core, have the dysfunction of WAT in common [7]. Specifically, within “unhealthy” WAT there is increased release of inflammatory markers, mitochondrial dysfunction, and increased oxidative stress due to lack of adequate vascular perfusion of WAT in obese patients [8,9]. Recently, the ability to induce WAT beiging has been highly regarded as a method to improve vascularization of adipose tissue through angiogenesis as well as improvements in mitochondrial function as potential modalities to reduce obesity-related diseases and mortality.

The endocannabinoid system (ECS) plays a critical role in the control of energy homeostasis. The ECS increases uptake and storage of energy by acting through both central and peripheral mechanisms [10]. Chronic treatment with the CB1R antagonist rimonabant (RIM) leads to weight loss and increased insulin sensitivity in multiple models of obesity, including rodents [11,12], canines [13,14], and humans [15]. Weight loss was attributed to decreased fat mass as a result of a reduction in adipocyte cell size in dogs, despite maintenance on a high-fat diet [16]. In addition, it was shown in rodents that RIM reduced the lipid content of BAT and increased activation of thermogenesis and energy expenditure [17]. Recently, we reported the mechanism by which CB1R antagonism promotes WAT beiging [18]. β3-adrenergic (β3) and natriuretic peptide (NP) receptors in WAT depots were upregulated following treatment with RIM and coincided with increased lipolysis-induced beiging [19,20]. NP and NP receptors (NPR) have been shown to act as powerful lipolytic agents in human WAT in situ and in isolated fat cells in vitro [20,21,22]. Consistent with our canine data, the reduction of WAT mass in rodents by RIM resulted from enhanced lipolysis and increased energy expenditure due to in part to NP activation.

Since RIM crosses the blood–brain barrier, the drug exerts both central and peripheral pathways to facilitate its functions. Its central action has been linked to adverse effects which have limited the drug’s clinical application [23]. However, the peripheral CB1R antagonist, AM6545, has limited brain penetrance and has been shown to decrease food intake and reduce body weight [24]. In addition, peripheral CB1R blockade activates BAT and diminishes dyslipidemia and obesity [25]. Given the promise of peripheral CB1 antagonist AM6545, the aim of this study is to determine the cellular and molecular mechanisms by which peripheral CB1R antagonism acts on beiging, lipolysis, mitochondrial biogenesis, and oxygen consumption rate (OCR) when the central and sympathetic nervous system effects could be avoided.

## 2. Results

### 2.1. Treatment with CB1R Antagonists Does Not Affect Cell Viability

**Figure 1:**
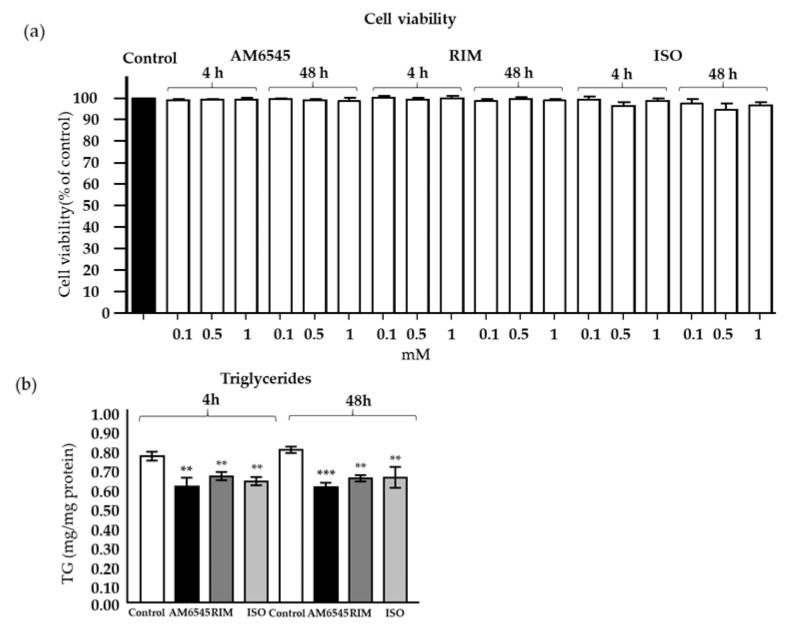
(**a**) Cell viability in 3T3-L1-differentiated cells were treated with AM6545, rimonabant (RIM), and isoproterenol (ISO) at 4 and 48 h. (**b**) Cell triglyceride (TG) content after 4 and 48 h of treatment. Data on graphs are presented as mean ± Standard Error of Mean SEM of 4 independent rounds of the cells; ** *p* < 0.01 vs. control *** *p* < 0.001 vs. control.

To assess any cytotoxic effects of AM6545, RIM, and isoproterenol (ISO), mature 3T3-L1 adipocytes were cultured with each drug at variable doses (0.1, 0.5, and 1 mM; Figure 1a). AM6545, RIM, and ISO did not cause cytotoxicity at 4 and 48 h at any of the concentrations tested. Based on a previous study from Watanabe et al. [26], 0.1–1 mM RIM increased adiponectin secretion and gene expression.

### 2.2. Treatment with CB1R Antagonists Decreased Triglyceride Content

Accumulation of triglycerides in AM6546-treated cells was similar to RIM- and ISO-treated cells and significantly blunted compared to vehicle (Figure 1b; *p* < 0.001).

### 2.3. The Peripheral CB1R Antagonist-Induced Genes Involved in Beiging in 3T3-L1 Mature Adipocytes

**Figure 2:**
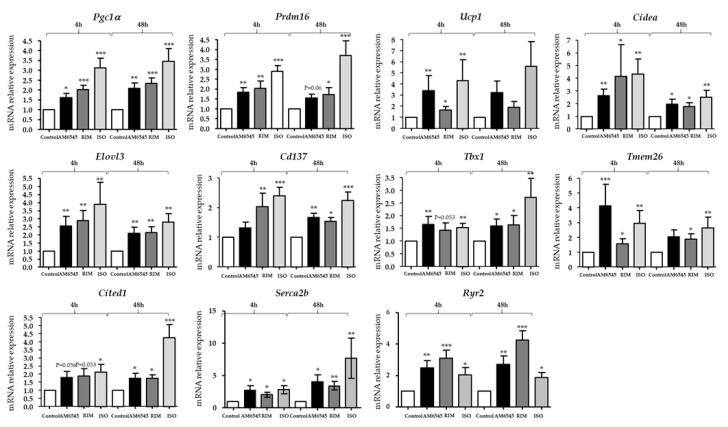
Peripheral cannabinoid receptor 1 (CB1R) antagonist increases markers of beiging in 3T3-L1 adipocytes. 3T3-L1-differentiated cells were treated with AM6545, rimonabant (RIM), and isoproterenol (ISO) for 4 and 48 h. Gene expression of beiging markers (*peroxisome proliferator-activated receptor gamma coactivator 1-alpha* (*Pgc1a*), *PR domain containing 16* (*Prdm16), uncoupling protein 1* (*Ucp1*), cell death-inducing DFFA-like effector A (*Cidea*), elongation of very long chain fatty acids protein 3 (*Elovl3*), tumor necrosis factor receptor superfamily member 9 (*Cd137*), *T-box transcription factor* (*Tbx1*), transmembrane protein 26 (*Tmem26*), cbp/p300-interating transactivator 1 (*Cited1), sarco/endoplasmic reticulum Ca^2+^-ATPase 2b* (*Serca2b*), and *ryanodine receptor 2* (*Ryr2*)) was evaluated by RT-PCR. Data on graphs are presented as mean ± SEM of 4 independent rounds of the cells; * *p* < 0.05 vs. control ** *p* < 0.01 vs. control *** *p* < 0.001 vs. control.

To examine the possible beiging effect of treatment with the peripheral CB1R antagonist, expression of key beiging markers was examined (Figure 2). The 3T3-L1 adipocytes were treated with 0.5 mM of AM6545 or RIM and 10 mM ISO. Consistent with previous findings in the adipose tissue [18], AM6545 significantly increased many of the genes involved in beiging of adipocytes including *Peroxisome proliferator-activated receptor gamma coactivator 1-alpha* (*Pgc1α*) (*p* < 0.05 and *p* < 0.01), *PR domain containing 16* (*Prdm16*) (*p* < 0.01 and *p* < 0.05), *uncoupling protein 1* (*Ucp1*) (*p* = 0.096 and *p* < 0.05), *cell* death-inducing *DNA fragmentation factor alpha*-like effector *A*(*Cieda*) (*p* < 0.01 and *p* < 0.05), *elongation of very long chain fatty acids protein 3* (*Elovl3*) (*p* < 0.016 and *p* < 0.05), *tumor necrosis factor receptor superfamily member 9* (*Cd137*) (*p* = 0.15 and *p* < 0.01), *T-box transcription factor* (*Tbx1*) (*p* < 0.05 and *p* < 0.05), *transmembrane protein* 26 (*Tmem26*) (*p* < 0.05 and *p* < 0.005), *cbp/p300-interacting transactivator 1* (*Cited1*) (*p* < 0.05 and *p* < 0.05), *sarco/endoplasmic reticulum Ca^2+^-ATPase 2b* (*Serca2b*) (*p* < 0.05 and *p* < 0.05), *ryanodine receptor 2* (*Ryr2*) (*p* < 0.05 and *p* < 0.01) at 4 and 48 h, respectively, compared to the vehicle.

### 2.4. Lipolysis Increased by the Peripheral CB1R Antagonist

**Figure 3:**
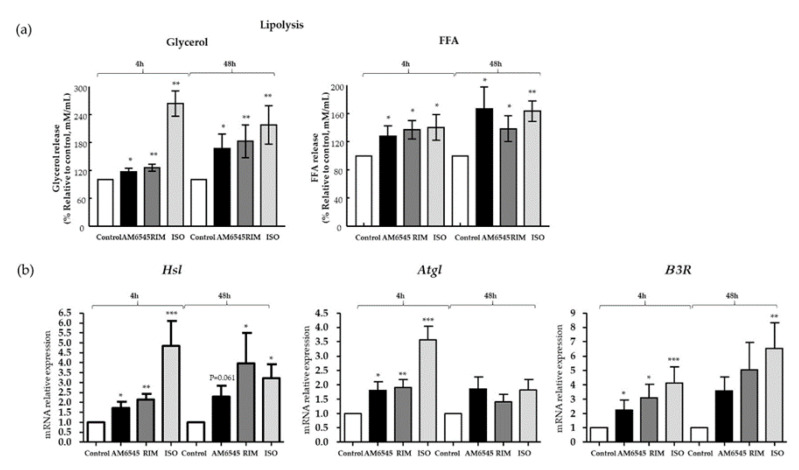
Peripheral cannabinoid receptor 1 (CB1R) antagonist increased lipolysis. 3T3-L1 adipocytes were treated with AM6545, rimonabant (RIM), and isoproterenol (ISO) for 4 and 48 h. (**a**) AM6545 increased glycerol and free fatty acid (FFA) releases into the media. (**b**) AM6545 increased genes involved in lipolysis such as *hormone-sensitive lipase* (*Hsl*), *adipose triglyceride lipase* (*Atgl*), and beta-3-adrenergic receptor (β3R). Data on graphs are presented as mean ± SEM of 4 independent rounds of the cells; * *p* < 0.05 vs. control ** *p* < 0.01 vs. control *** *p* < 0.001 vs. control.

It has been shown that RIM increases lipolysis in primary adipocytes from canines [18] and rats [27]. We hypothesized that the peripheral CB1R antagonist, AM6545, would enhance adipose tissue lipolysis, similar to RIM, and, therefore, facilitate fatty acid utilization as a mechanism associated with beiging. To test this, we determined glycerol and free fatty acid (FFA) release into the media as an indicator of lipolysis following treatment of adipocytes. Glycerol and FFA release following AM6545 treatment was higher than release in adipocytes treated with vehicle (*p* < 0.05 and *p* < 0.05, at 4 and 48 h, respectively) and comparable to treatment with RIM and ISO (Figure 3a). Key genes involved in lipolysis such as *hormone sensitive lipase* (*Hsl*); *p* < 0.05 and *p* = 0.061), *adipose triglyceride lipase* (*Atgl*); *p* < 0.05 and *p* = 0.1), and *β3-adrenergic receptor* (*β3R*; *p* < 0.05 and *p* = 0.08) increased significantly after 4 and 48 h of treatment, respectively, in 3T3-L1 cells treated with AM6545. These increases are similar to what was seen after treatment with RIM (Figure 3b).

### 2.5. The Peripheral CB1 Antagonist Increased mtDNA and Mitochondrial Biogenesis Genes

**Figure 4:**
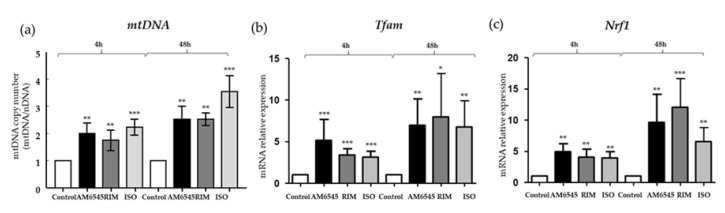
Peripheral cannabinoid receptor 1 (CB1R) antagonist increased mitochondrial DNR (mtDNA) and mitochondrial biogenesis genes. 3T3-L1 adipocytes were treated with AM6545, RIM, and ISO for 4 and 48 h. (**a**) AM6545, rimonabant (RIM), and isoproterenol (ISO) increased mtDNA. (**b**,**c**) Markers of mitochondrial biogenesis, *transcription factor A, mitochondrial* (*Tfam*), and *nuclear respiratory factor 1* (*Nrf1*), were also increased following treatment with AM6545, RIM, and ISO. Data on graphs are presented as mean ± SEM of 4 independent rounds of the cells; * *p* < 0.05 vs. control ** *p* < 0.01 vs. control *** *p* < 0.001 vs. control.

mtDNA copy numbers were measured in 3T3-L1 adipocytes treated with AM6545, RIM, and ISO, and compared to the vehicle. The mtDNA/nDNA ratio of AM6545, RIM, and ISO increased significantly by 2.0-, 1.8-, and 2.2-fold (*p* < 0.01, *p* < 0.01, and *p* < 0.001, respectively) after 4 h of treatment. AM6545, RIM, and ISO mtDNA/nDNA ratio increased by 2.5- and 3.5-fold (*p* < 0.01, *p* < 0.01, and *p* < 0.001, respectively) after 48 h of treatment (Figure 4a). In addition, we measured genes involved in mitochondrial biogenesis such as *transcription factor A, mitochondrial* (*Tfam)*, and nuclear *respiratory factor 1* (*Nrf1)* after 4 and 48 h of treatment with AM6545, RIM, and ISO in 3T3-L1 adipocytes. After 4 h of treatment, *Tfam* was increased by 5.2-fold in AM6545 (*p* < 0.001), by 3.4-fold in RIM (*p* < 0 < 0.001), and by 3.1-fold ISO (*p* < 0.001). AM6545, RIM, and ISO Tfam gene expression increased by 6.9-, 8.0-, and 6.8-fold (*p* < 0.01, *p* < 0.05, and *p* < 0.01, respectively) after 48 h of treatment (Figure 4b). After 4h, AM6545, RIM, and ISO increased *Nrf1* gene expression by 4.9-, 4.0-, and 3.9-fold (*p* < 0.01, *p* < 0.01, and *p* < 0.01, respectively). AM6545, RIM, and ISO increased Nrf1 expression at 48 h by greater than 8-fold (*p* < 0.01, *p* < 0.001, and *p* < 0.01, respectively) (Figure 4c).

### 2.6. The Peripheral CB1R Antagonist Increased Mitochondrial Respiration and Proton Leak in 3T3-L1 Adipocytes

**Figure 5:**
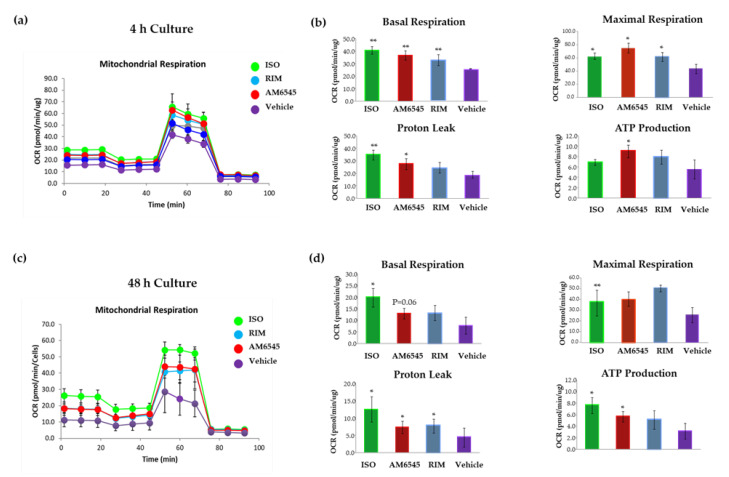
Peripheral cannabinoid receptor 1 (CB1R) antagonist increased oxygen consumption rate (OCR). 3T3-L1 adipocytes were treated with AM6545, rimonabant (RIM), and isoproterenol (ISO) at 4 and 48 h. OCR was measured in basal conditions or in response to sequential treatment with 2 oligomycin, 0.75 FFCP (respiratory chain uncoupler), and 1 µM rotenone/antimycin A (inhibitor of respiratory chain complex I and complex III) using Seahorse XF-24 analyzer. (**a**) Mitochondrial respiration curves at 4 h after treatment. (**b**) Parameters calculated from the tracing at 4 h after treatment. (**c**) Mitochondrial respiration curves 48 h after treatment. (**d**) Parameters calculated from the OCR at 48 h after treatment. Data on graphs are presented as mean ± SEM of e4 independent rounds of the cells; * *p* < 0.05 vs. control ** *p* < 0.01 vs. control.

Given the increase in mitochondrial biogenesis following treatment with the CB1R antagonists, we next explored the effects of CB1R antagonism on mitochondrial respiration. The OCR was measured in mature 3T3-L1 adipocytes. The OCR curves at 4 and 48 h are presented in Figure 5a,c. Cells treated with AM6545 at 4 and 48 h had higher oxygen consumption than the vehicle group. Similarly, OCR increased after 4 and 48 h culture in cells treated with RIM and ISO.

As shown in Figure 5b, after 4 h of treatment with AM6545, basal respiration (*p* < 0.01), maximal respiration (*p* < 0.05), proton leak (*p* < 0.05), and ATP synthesis (*p* < 0.05) increased about 1.7-fold compared to the vehicle. At 48 h (Figure 5d), basal mitochondrial respiration of AM6545-treated adipocytes increased by 1.3-fold (*p* = 0.06). Proton leak increased 1.3-fold (*p* < 0.05) and ATP production increased by 1.4-fold (*p* < 0.05) compared to the control. Maximal respiration did not change significantly. Similar results at both time points were observed with RIM. ISO showed higher OCR compared to the vehicle and the CB1R antagonists at 4 and 48 h.

### 2.7. CB1R Antagonism-Induced Improvements in Mitochondrial Function Rely on Lipolysis, as Shown During Real-Time OCR

**Figure 6:**
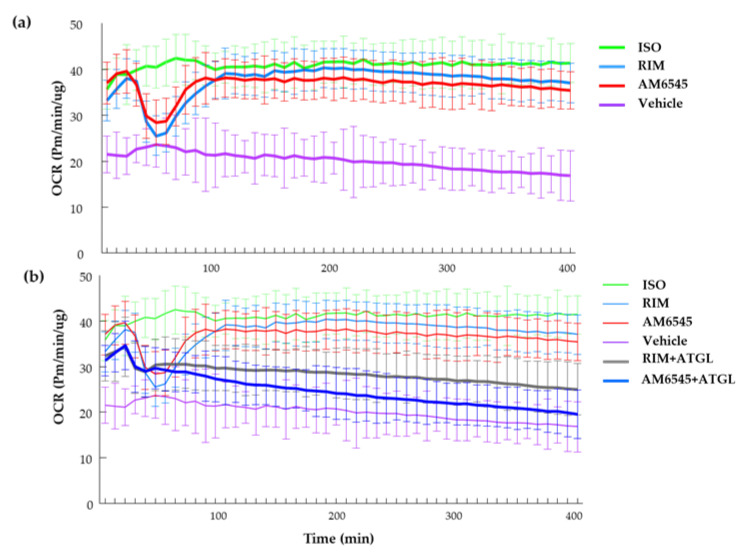
Real-time oxygen consumption rate (OCR) and effect of lipolysis. (**a**) Time course after differentiated 3T3-L1 treated with AM6545, rimonabant (RIM), isoproterenol (ISO), and vehicle. (**b**) Time course of treatments including the use of Atglinstatin with AM6545 and RIM. Data on graphs are presented as mean ± SEM of 4 independent rounds of the cells.

We next evaluated the real-time OCR effects of the CB1R antagonists on 3T3-L1 adipocytes. Unexpectedly, during the first 45 min of treatment with AM6545 and RIM, OCR decreased compared to both ISO and the vehicle (Figure 6a). The OCR of the CB1R-treated cells then increased and was similar to ISO treatment for the remainder of the analysis. We hypothesized that the initial drop in OCR was due to a change in substrate utilization of the cells followed by a subsequent use of fatty acids due to increased lipolysis. To test this, we used an ATGL inhibitor, Atglinstatin, to evaluate the association between the temporary decrease in OCR and lipolysis. ATGL inhibition prevented AM6545- and RIM-induced OCR drops (Figure 6b), suggesting the immediate effects of CB1R antagonists on inducing lipolysis.

**Figure 7:**
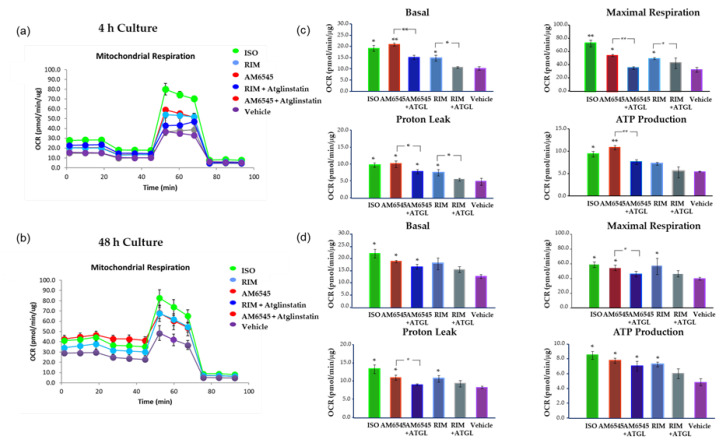
Peripheral cannabinoid receptor 1 (CB1R) antagonist increased oxygen consumption rate (OCR) inhibited by lipolysis blocker. 3T3-L1 adipocytes were treated with AM6545, rimonabant (RIM) with and without Atglinstatin, and isoproterenol (ISO) at 4 and 48 h. OCR was measured in basal conditions or in response to sequential treatment with 2 µM oligomycin, 0.75 µM FFCP (respiratory chain uncoupler), and 1 µM rotenone/antimycin A (inhibitor of respiratory chain complex I and complex III) using Seahorse XF-24 analyzer. (**a**) Mitochondrial respiration tracing using Seahorse at 4 h after treatment. (**b**) Parameters calculated from the tracing at 4 h after treatment. (**c**) Mitochondrial respiration tracing 48 h after treatment. (**d**) Parameters calculated from the tracing at 48 h after treatment. Data on graphs are presented as mean ± SD of 4 independent rounds of the cells; * *p* < 0.05 vs. control ** *p* < 0.01 vs. control.

To evaluate the role of lipolysis on CB1R antagonist-induced changes to OCR, we treated 3T3-L1 adipocytes with AM6545 and RIM with and without Atglinstatin for 4 and 48 h (Figure 7). Consistent with previous results, the OCR of AM6545 treatment without Atglinstatin increased significantly compared to the vehicle. Treatment with Atglinstatin decreased the OCR of the cells treated with both AM6545 and RIM after 4 and 48 h in culture. Basal respiration and ATP production were significantly decreased at 4 h when cells were prevented from undergoing lipolysis during treatment with AM6545 (*p* < 0.01 and *p* < 0.05, respectively; Figure 7b) compared to AM6545 treatment alone. Similar results were found at 4 and 48 h (Figure 7d) for maximal respiration (*p* < 0.01 and *p* < 0.05, respectively) and proton leak (*p* < 0.05, for both time points) when cells were treated with AM6545 and Atglinstatin compared to AM6545 alone. Similar results were observed with RIM and Atglinstatin compared to RIM alone at 4 h.

## 3. Discussion

Peripheral CB1R antagonists are gaining attention for their therapeutic use in obesity and related diseases. The peripheral CB1R antagonist AM6545 has been shown to have limited brain penetrance and, yet, is still efficacious in reducing body weight and improving dyslipidemia in insulin resistant mice [28,29,30]. Our results are consistent with these findings. In addition, we provide a mechanism by which this peripheral CB1R antagonist increased markers of beiging and OCR, via upregulation of lipolysis. Importantly, the beneficial effects of the direct application of peripheral CB1R antagonist on adipocytes are similar to those seen with brain-penetrating CB1R antagonists, suggesting that the central effects of CB1R antagonist could be avoided while still delivering metabolic benefits.

Recently, we demonstrated that RIM promotes beiging of subcutaneous and visceral fat depots in fat-fed dogs [18]. Our current findings suggest that AM6545 promotes upregulation of beiging markers and improves mitochondrial function in 3T3-L1 adipocytes similar to what we observed following in vivo administration of RIM. In the current study, key beiging genes such as *Pgc1α, Prdm16, Cidea, Elovl3, Cd137, Tmem26, Tbx1,* and *Cited1* were increased following treatment with AM6545 and RIM, with larger increases in *Ucp1* seen with AM6545 than were seen with in vivo or in vitro treatment with RIM, suggesting that mitochondrial uncoupling may be greater following treatment with the non-brain-penetrating antagonist.

Serca2b and Ryr2 are important genes in futile calcium cycling in the mitochondria and can increase thermogenesis independent of *Ucp1* [31]. We have previously shown that these genes are upregulated following treatment with RIM in fat-fed dogs, with significant but minimal increases in *Ucp1* [18]. In our current study, AM6545 treatment increased expression of both Serca2b and Ryr2 at both 4 and 48 h of treatment. RIM had similar, if not greater, increases in expression at both time points. While some UCP1-independent pathways are only active when *Ucp1* is downregulated, no such dynamic has been shown for futile calcium cycling [32]. There are, in fact, a number of examples of alternative thermogenesis pathways that do not require the inactivation of UCP1 [33,34]. Given our previous data, it stands to reason that both futile calcium cycling and UCP1-induced uncoupling can both contribute to thermogenesis and beiging simultaneously, either in the same cells or in different cells within the same population. This latter option seems more likely, given the heterogeneity of adipose tissue [31,35].

Once beiging machinery increases, the thermogenic action of mitochondria requires increased fuel such as FFA [36,37]. To supply the fuel, lipolysis is stimulated within the adipocytes [19]. Specifically, AM6545 increases lipolysis, demonstrated through the release of glycerol and FFA into the culture media after 4 and 48 h of cell culture. Similar results were observed with RIM and the positive control, ISO. AM6545 increased expression of *Hsl, Atgl,* and *β3R* and decreased TG storage, further demonstrating its effect on lipolysis.

Increased lipolysis can be stimulated by increases in NPR and β3R [18]. In addition, β3R has a critical role in thermogenesis. β3R stimulation by pharmacological agonists, such as CL316,243 and ISO, induces higher thermogenic capacity in WAT [38]. Our data suggest that β3R is upregulated by AM6545. Taken together with the induction of beiging machinery, AM6545 appears to increase thermogenic capacity of WAT.

β3R stimulation also increases *Pgc1α* expression [39] and leads to increases in UCP1 via the activation of several nuclear and non-nuclear receptor factors [40]. PGC1α plays a number of important roles in regulating metabolism, including the regulation of mitochondrial oxidative phosphorylation and muscle fiber-type switching [41]. Its major role in mitochondrial biogenesis [42] and thermogenesis has made eα a target for anti-obesity therapy. Our data suggest that AM6545, similar to RIM and the β3 agonist ISO, increases *Pgc1α* expression via *β3R* activation, leading to increased mitochondrial biogenesis, similar to what has been previously shown in other mouse cell lines following catecholamine activation [43]. Consistent with these results we showed that mtDNA and genes related to mitochondrial biogenesis, such as *Tfam* and *Nrf1*, were significantly upregulated. The increased mitochondrial biogenesis was corroborated by increases in oxygen consumption and proton leak of adipocytes treated with AM6545. Importantly, the increased mitochondria within the adipocytes may also contribute to increased insulin sensitivity [44,45].

Treatment with the peripheral CB1R antagonist significantly increased OCR by increasing basal and maximal respiration as well as proton leak and ATP production at 4 and 48 h. Unexpectedly, AM6545 and RIM showed a transient decrease of real time OCR during the first 45 min after treatment initiation. This effect was not seen in ISO-treated cells. Following this dip, the OCR increased to match the OCR levels seen in ISO-treated cells over the course of the remainder of the 400-min study. This transient dip in oxygen consumption was due to CB1R antagonist-induced lipolysis, as treatment with the lipolysis blocker Atglinstatin reversed the effects of AM6545 and RIM on OCR during the first 45 min of treatment. In the presence of Atglinstatin, OCR for cells treated with AM6545 and RIM remained no different than vehicle control for the entire study duration. Atglinstatin also decreased basal respiration, maximal respiration, proton leak, and ATP production, most importantly after 4 h treatment with CB1R antagonists, further suggesting that the impact of CB1R antagonism on adipocytes is driven by increased lipolysis.

Taken together, our results reveal that peripheral CB1R antagonist AM6545 enhances the beiging process and mitochondrial function via lipolysis in adipocytes similarly to the brain-penetrating CB1R antagonist RIM. Our data provide a potential mechanism by which physiologic responses and improvement of energy expenditure, lipid profiles, and insulin sensitivity are improved following dosing with peripheral CB1R antagonists in previous in vivo studies [28,46,47]. We acknowledge our data solely focus on an in vitro model but provide a beginning point to understand the mechanism by which this compound may improve adipose tissue function. We submit that further in vivo studies in knockout and large animal models, followed by studies in humans, are required to elucidate the potential applications of peripheral CB1R antagonism as a therapeutic agent for obesity.

The CB1R antagonist RIM demonstrated marked improvements in obesity, insulin resistance, and other metabolic perturbations in patients. However, its serious side-effect profile made it unsafe for patients. Here, we suggest that a peripherally-restricted CB1R antagonist, AM6545, increases adipocyte beiging and improves mitochondrial function via increased lipolysis. We cannot discard other metabolic pathways such as reduced inflammation or reduced endocannabinoids to be involved in the beneficial effects of the peripheral CB1R on adipocytes. Thus, a direct role for CB1R antagonism on adipocytes does not require brain penetrance, supporting the importance of pursuing peripheral CB1R antagonism for the pharmacological treatment of obesity, T2D, and related metabolic diseases. Future studies are needed to verify the effects in larger animal models and in patients.

## 4. Materials and Methods

### 4.1. Preparation and Treatment of 3T3-L1 Adipocytes

Mouse embryo 3T3-L1 preadipocytes (American Type Culture Collection (ATCC), Manassas, VA, USA) were maintained in DMEM/F-12 (ATCC, Manassas, VA, USA) supplemented with 10% bovine calf serum (ATCC, Manassas, VA, USA) and 1% penicillin-streptomycin (Thermo Fisher Scientific, Waltham, MA, USA) until confluent (48–72 h). As performed in Miller et al. [48], the differentiation was induced in DMEM/F-12 media containing 10% fetal bovine serum (Thermo Fisher Scientific), 5 uM dexamethasone (Sigma-Aldrich, St. Louis, MO, USA), 0.5 μg/mL insulin (Sigma-Aldrich), 0.5 mM isobutylmethylxanthine (Sigma-Aldrich), 1 μM rosiglitazone (Sigma-Aldrich), and 1 nM T3 (Sigma-Aldrich) for 4 days. Cells were then differentiated in DMEM/F-12 media supplemented with 10% fetal bovine serum, 0.5 μM insulin, and 1 nM T3 for 3 additional days. After 7 days, all the cells were matured, and we treated with 0.5 mM rimonabant (Sigma-Aldrich), 0.5 mM AM6545 (kindly donated by Dr. Makriyannis from Northeastern University Center for Drug Discovery, Boston, MA, USA), 10 mM isoproterenol (ISO) (Sigma-Aldrich); ISO was used as a positive control, as described [48], and vehicle. The dose of RIM was chosen based on a previous study from Watanabe et al. [26], where they showed that 0.1–1 μM RIM increased adiponectin secretion and gene expression. A similar dose was chosen for AM6545 to be comparable with RIM. We followed the protocol published by Miller et al. [48], ISO increased OCR, and beiging genes, and we used the same protocol to compare CB1R antagonist treatments; therefore, the cells were incubated for 4 h (*n* = 2–3 per condition/4 independent rounds) and 48 h (*n* = 2–3 per condition/4 independent rounds).

### 4.2. Cell Viability and Triglyceride Measurements

Lactate dehydrogenase (LDH) levels in culture medium were determined to assess cell toxicity by a commercially available ELISA kit (Roche Applied Science, Indianapolis, IN, USA). The percentage of viable cells was calculated by defining the cell viability without treatment as 100% viability. 3T3-L1 cell lysates in 1% Triton X-100 in PBS were collected for triglyceride (TG) measurements. A TG assay kit (Sigma–Aldrich) was used according to the method as described previously [49]. Cellular TG content was then normalized to the protein concentration as measured by a BCA protein assay kit (Bio-Rad, Hercules, CA, USA). Results are represented as the amount of TG in mg to an equivalent of cellular proteins (in mg).

### 4.3. Lipolysis Assays

Samples of the medium were collected and measured for glycerol and FFA release. The glycerol assay kit (Sigma-Millipore, St. Louis, MO, USA) was used in accordance with the manufacturer’s instructions. FFA were measured using the NEFAC colorimetric assay in accordance with the manufacturer’s instructions (Wako Pure Chemical Industries, Richmond, VA, USA).

### 4.4. Mitochondrial DNA Copy Number

The ratio of mtDNA to nuclear DNA in 3T3-L1 adipocytes was reflecting the cellular mitochondrial number, and was determined by RT-PCR as previously described [50]. DNA was isolated by QIAamp DNA mini-kit (Qiagen, Valencia, CA, USA). The abundance of the mitochondrial DNA (mtDNA) was evaluated by measuring nicotinamide adenine dinucleotide dehydrogenase 1 gene (Nd1) (primer pairs: Forward: 5′-ACCATTTGCAGACGCCATAA-3′, reverse: 5′-TGAAATTGTTTGGGCTACGG-3′), using the LightCycler FastStart DNA Master SYBR Green I (Roche Applied Science, Indianapolis, IN, USA). mtDNA content was normalized to 18S rRNA gene (primer pairs: Forward: 5′-TAGAGGGACAAGTGGCGTTC-3′, reverse: 5′-CGCTGAGCCAGTCAGTGT-3′. Reactions were incubated at 95 °C for 10 min, then 45 cycles of 95 °C for 10 s, 62 °C for 20 s, and 72 °C for 20 s.

### 4.5. Measurement of Oxygen Consumption Rate

3T3-L1 pre-adipocytes were seeded at a density of 15,000 cells/well to the to the XFe24 microplate (e, Agilent Technologies, Santa Clara, CA, USA) coated with 0.2% gelatin, and cells were differentiated as described above. For OCR measurements after 4 and 48 h of exposure to the various drugs to the mature adipocytes, cells were exposed to the same doses that we used for beiging experiments (0.5 mM rimonabant (Sigma-Aldrich), 0.5 mM AM6545 (Northeastern University Center for Drug Discovery, Boston, MA, USA), 10 mM isoproterenol (Sigma-Aldrich), used as a positive control, as described previously [48], and a vehicle). Cells were washed 3 times with XF Assay medium containing 4.5 g/L glucose, 4.0 mM glutamine, and 1.0 mM sodium pyruvate (pH was adjusted to 7.35 ± 0.05 using 1 M NaOH). The plates were placed in a 37 °C incubator without CO_2_ for one hour prior to the assay. OCR measurements were performed using Seahorse Biosciences XF Analyzer (Agilent Technologies) during basal conditions or in response to sequential treatment with 2 oligomycin (to block ATP synthesis), 0.75 FFCP (respiratory chain uncoupler), and 1 µM rotenone/antimycin A (inhibitor of respiratory chain complex I and complex III).

To determine the direct effects of AM6545 and RIM on the adipocytes in real-time, cells were treated with the same dose as described above via an injection port (6 replicates per drug). OCR was measured every 8.5 min for 400 min. To demonstrate the effect of lipolysis we added 20 μM ATGL inhibitor Atglinstatin (Sigma-Aldrich) as described previously [51], with 0.5 AM6545 or 0.5 mM RIM. The real-time measurements were performed for 400 min.

### 4.6. Total RNA Isolation and Real Time PCR (RT-PCR)

RNA was extracted from cell lysates using the Tri-Reagent^®^ Kit (Molecular Research Center, Cincinnati, OH, USA). First-strand cDNA was synthesized, per the manufacturer’s protocol, from 1 µg of total RNA obtained using Superscript II (Invitrogen, Carlsbad, CA, USA). RT-PCR was performed using a Light-Cycler 480 instrument (Roche Applied Science, Indianapolis, IN, USA). cDNA was amplified using on a Roche microplate with a final volume of 10 µL reaction mix containing 2.5 µL of 100-fold diluted cDNA, 7 µL LightCycler TaqMan Master Mix buffer (Roche Probes Master kit, Roche Applied Science, Indianapolis, IN, USA), and 0.5 µL specific TaqMan probes from Thermo Fisher Scientific (Table 1). Reactions were incubated at 95 °C for 10 min, then 45 cycles of 95 °C for 10 s, 60 °C for 30 s, then 72 °C for 2 s. *Mouse β-actin* (*Actb*) was used as the reference gene. Data was normalized and relative expression was determined from the threshold cycle (Ct) following the 2^−ΔΔCT^ method.

### 4.7. Statistical Analysis

All data were plotted as mean ± SEM using STATA (STATA 16MP, StataCorp LLC, College Station, TX, USA). The Kruskal–Wallis equality-of-populations rank test was used to perform the multiple comparison between the various groups. This analysis was followed by Dunnett’s test for pairwise comparisons. A *p*-value as indicated was considered statistically significant: * *p* < 0.05; ** *p* < 0.01; *** *p* < 0.001.

## Figures and Tables

**Table 1:** List of TaqMan^®^ primers.

*PGC1a*	Mm01,208,835_m1
*Prdm16*	Mm00,712,556_m1
*Ucp1*	Mm01,244,861_m1
*Cidea*	Mm00,432,554_m1
*Elovl3*	Mm00,468,164_m1
*Tbx1*	Mm00,448,949_m1
*Cd137*	Mm00,441,899_m1
*Tmem26*	Mm01,173,641_m1
*Cited1*	Mm01,235,642_g1
*Serca2b*	Mm01,201,431_m1
*Ryr2*	Mm00,465,877_m1
*ATGL*	Mm00,503,040_m1
*HSL*	Mm00,495,359_m1
*b3R*	Mm02,601,819_g1
*Tfam*	Mm00,447,485_m1
*Nrf*	Mm01,135,607_m1
*Nd1*	Mm04,225,274_s1
*Actb*	Mm02,619,580_g1

## Abbreviations

CB1R	Cannabinoid receptor 1
RIM	Rimonabant
ISO	Isoproterenol
OCR	Oxygen consumption rate
T2D	Type 2 diabetes
WAT	White adipose tissue
BAT	Brown adipose tissue
ECS	Endocannabinoid system
β3	Beta-3 adrenergic
NP	Natriuretic peptide
Pgc1α	Peroxisome proliferator-activated receptor gamma coactivator 1-alpha
Prdm16	PR domain containing 16
Ucp1	Uncoupling protein 1
Cidea	Cell death-inducing DFFA-Like effector A
Elovl3	elongation of very long chain fatty Acids protein 3
Cd137	tumor necrosis factor receptor superfamily member 9
Tbx1	T-box Transcription factor
Tmem26	Transmembrane protein 26
Cited1	Cbp/p300-interacting transactivator 1
Serca2b	Sarco/endoplasmic reticulum Ca^2+^-ATPase2b
RyR2	Ryanodine receptor 2
ATGL	Adipose triglyceride lipase
HSL	Hormone sensitive lipase
FFCP	Carbonyl cyanide-4(trifluoromethoxy) phenylhydrazone
Tfam	transcription factor A, mitochondria
Nrf1	nuclear respiratory factor 1
Nd1	dinucleotide dehydrogenase 1
Actb	mouse β-actin
