# Correction: Paszkiewicz et al. A Peripheral CB1R Antagonist Increases Lipolysis, Oxygen Consumption Rate, and Markers of Beiging in 3T3-L1 Adipocytes Similar to RIM, Suggesting That Central Effects Can Be Avoided. *Int. J. Mol. Sci.* 2020, *21*, 6639

**DOI:** 10.3390/ijms22094366

**Published:** 2021-04-22

**Authors:** Rebecca L. Paszkiewicz, Richard N. Bergman, Roberta S. Santos, Aaron P. Frank, Orison O. Woolcott, Malini S. Iyer, Darko Stefanovski, Deborah J. Clegg, Morvarid Kabir

**Affiliations:** 1 Sports Spectacular Diabetes and Obesity Wellness and Research Center, Cedars-Sinai Medical Center, Los Angeles, CA 90048, USA; RPaszkiewicz@mednet.ucla.edu (R.L.P.); Richard.Bergman@cshs.org (R.N.B.); santosrds1@gmail.com (R.S.S.); aaronpfrank@gmail.com (A.P.F.); Orison.Woolcott@gmail.com (O.O.W.); malini.s.iyer@gmail.com (M.S.I.); 2 School of Veterinary Medicine, University of Pennsylvania, Philadelphia, PA 19104, USA; sdarko@vet.upenn.edu; 3 The College of Nursing and Health Professions, Drexel University, Philadelphia, PA 19104, USA; djc387@drexel.edu

The authors wish to make the following corrections to this paper [1]: On page 6, the curve in Figure 5a was switched with Figure 7c on Page 7. Thus, Figure 5 should be replaced with the following figure (Figure 1), and Figure 7 with the following figure (Figure 2).

The authors apologize for any inconvenience caused and state that the scientific conclusions are unaffected. The original article has been updated.

## Figures and Tables

**Figure 1:**
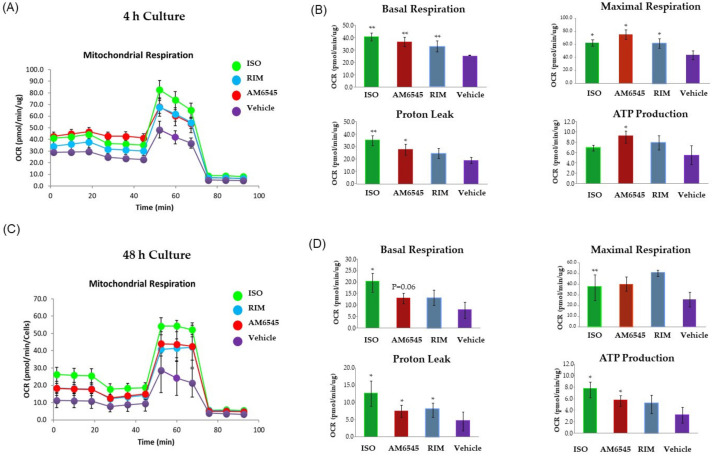
Peripheral cannabinoid receptor 1 (CB1R) antagonist increased oxygen consumption rate (OCR). 3T3-L1 adipocytes were treated with AM6545, rimonabant (RIM), and isoproterenol (ISO) at 4 and 48 h. OCR was measured in basal conditions or in response to sequential treatment with 2 oligomycin, 0.75 FFCP (respiratory chain uncoupler), and 1 µM rotenone/antimycin A (inhibitor of respiratory chain complex I and complex III) using the Seahorse XF-24 analyzer. (**A**) Mitochondrial respiration curves at 4 h after treatment. (**B**) Parameters calculated from the tracing at 4 h after treatment. (**C**) Mitochondrial respiration curves 48 h after treatment. (**D**) Parameters calculated from the OCR at 48 h after treatment. Data on graphs are presented as the mean ± standard error of mean (SEM) of 4 independent rounds of the cells; * *p* < 0.05 vs. control, ** *p* < 0.01 vs. control.

**Figure 2:**
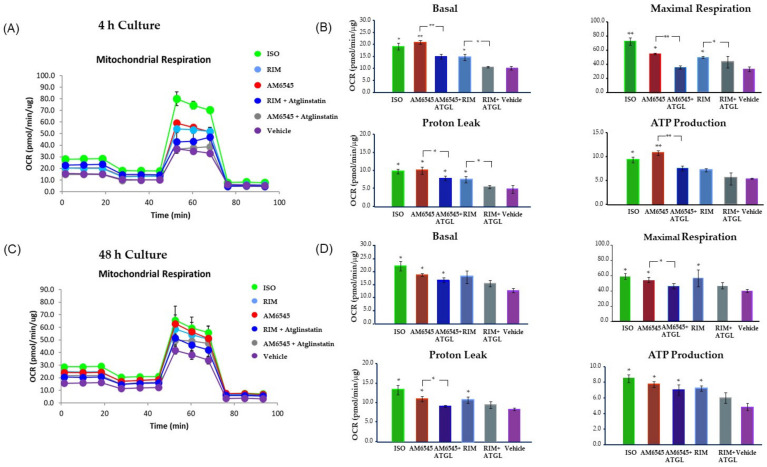
Peripheral cannabinoid receptor 1 (CB1R) antagonist increased oxygen consumption rate (OCR) inhibited by lipolysis blocker. 3T3-L1 adipocytes were treated with AM6545 and rimonabant (RIM) with and without Atglinstatin, and isoproterenol (ISO) at 4 and 48 h. OCR was measured in basal conditions or in response to sequential treatment with 2 µM oligomycin, 0.75 µM FFCP (respiratory chain uncoupler), and 1 µM rotenone/antimycin A (inhibitor of respiratory chain complex I and complex III) using the Seahorse XF-24 analyzer. (**A**) Mitochondrial respiration tracing using Seahorse at 4 h after treatment. (**B**) Parameters calculated from the tracing at 4 h after treatment. (**C**) Mitochondrial respiration tracing 48 h after treatment. (**D**) Parameters calculated from the tracing at 48 h after treatment. Data on graphs are presented as the mean ± standard deviation (SD) of 4 independent rounds of the cells; * *p* < 0.05 vs. control, ** *p* < 0.01 vs. control.
